# 

**Published:** 1894-10

**Authors:** 


### TABLE OF CONTENTS.

ORIGINAL COMMUNICATIONS. PAGE

An Artificial Nose. By P. W. Moriarity, D.M.D., Boston, Mass. . . . 609

Review and Revision of Practice. By S. B. Palmer, M.D.S., Syracuse, N. Y. . . . . 610

Metallic *versus* the Plastic Base for Artificial Dentures. By I. Norman Broomell, D.D.S., Philadelphia . . . . 620

The Editorial Function. By Henry Burchard, M.D., D.D.S., Philadelphia . . . . 624

Method of manipulating Gold as practised Fifty Years Ago. By F. B. Smith, M.D., New York City . . . . 627

Replantation in Chronic Alveolar Abscess. By Geo. A. Sullivan, D.D.S., Albany, N. Y. . . . . 628

Hemorrhages after Extraction of Teeth in Hemorrhagic Diathesis and Spontaneous Anæmia. By D. A. Rosenthal, D.D.S., Philadelphia . . . . 629

REPORTS OF SOCIETY MEETINGS.

New York Odontological Society . . . . 633

Odontological Society of Pennsylvania . . . . 644

Harvard Odontological Society . . . . 651

National Association of Dental Faculties . . . . 652

American Dental Society of Europe . . . . 657

National Association of Dental Examiners . . . . 658

EDITORIAL.

Are we degenerating? . . . . 662

BIBLIOGRAPHY . . . . 665

OBITUARY.

Dr. Ransom Walker . . . . 668

CURRENT NEWS.

New England Dental Society . . . . 670

Recent Patents . . . . 670

Union Dental Convention . . . . 671

SELECTIONS.

Guaiacol . . . . 671

